# Resolving Li‐Ion Battery Electrode Particles Using Rapid Lab‐Based X‐Ray Nano‐Computed Tomography for High‐Throughput Quantification

**DOI:** 10.1002/advs.202000362

**Published:** 2020-04-30

**Authors:** Thomas M. M. Heenan, Alice V. Llewellyn, Andrew S. Leach, Matthew D. R. Kok, Chun Tan, Rhodri Jervis, Dan J. L. Brett, Paul R. Shearing

**Affiliations:** ^1^ Electrochemical Innovation Lab, Department of Chemical Engineering UCL London WC1E 7JE UK; ^2^ The Faraday Institution, Quad One Harwell Science and Innovation Campus Didcot OX11 0RA UK

**Keywords:** electrodes, Li‐ion, metallurgy, powder, X‐ray CT

## Abstract

Vast quantities of powder leave production lines each day, often with strict control measures. For quality checks to provide the most value, they must be capable of screening individual particles in 3D and at high throughput. Conceptually, X‐ray computed tomography (CT) is capable of this; however, achieving lab‐based reconstructions of individual particles has, until now, relied upon scan‐times on the order of tens of hours, or even days, and although synchrotron facilities are potentially capable of faster scanning times, availability is limited, making in‐line product analysis impractical. This work describes a preparation method and high‐throughput scanning procedure for the 3D characterization of powder samples in minutes using nano‐CT by full‐filed transmission X‐ray microscopy with zone‐plate focusing optics. This is demonstrated on various particle morphologies from two next‐generation lithium‐ion battery cathodes: LiNi_0.8_Mn_0.1_Co_0.1_O_2_ and LiNi_0.6_Mn_0.2_Co_0.2_O_2_; namely, NMC811 and NMC622. Internal voids are detected which limit energy density and promote degradation, potentially impacting commercial application such as the drivable range of an electric vehicle.

## Graphical abstract

This work describes a fast preparation method and high‐throughput scanning procedure for the 3D characterization of lithium–ion battery electrode powder in minutes. This employs nanocomputed tomography by full‐filed transmission X‐ray microscopy with zone‐plate focusing optics. The procedure is outlined and subsequently used to qualitatively and quantitatively assess two next‐generation nickel‐rich cathode powders.

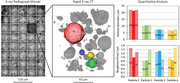

## 1 Introduction

Global production of powders is on the order of millions of tons per year. However, due to the intimate link between powder morphology and performance, many industries are required to not only produce very large quantities, but also to adhere to strict microstructural standards.^[^ ^1^ ^]^ The performance of the powder, once manufactured into a commercial product, is dictated by the application: Bone implants require sufficient mechanical integrity otherwise they may necessitate early replacement, causing further risk to the patient. Therefore, structural defects, such as cracked particles, can pose significant issues.^[^ ^2^ ^]^ Catalyst particles may be sized accordingly in order to produce sufficient reaction activity, or else the reaction production rate may decline, altering the profitability of the plant.^[^ ^3^ ^]^ Electrode particles within a lithium‐ion battery (LIB), require chemical and morphological consistency, in electric vehicles (EV), a defective electrode may result in an insufficient drivable range, or the premature replacement of the battery pack.^[^ ^4^ ^]^ These are three, of many, examples whereby the performance of the particles during commercial application is dictated significantly by the quality of the manufacturing process. Quality assurance (QA) methods which are capable of efficaciously assessing the particle microstructure, fast enough to be representative of commercial manufacturing rates, are vital.

X‐ray computed tomography (CT) has revolutionized the field of 3D microstructural analysis.^[^ ^5^, ^6^ ^]^ The rapid development of X‐ray tubes and zone‐plate focusing optics has produced lab‐based systems capable of demonstrating comparable imaging quality to specialist synchrotron facilities.^[^ ^6^, ^7^, ^8^, ^9^, ^10^ ^]^ Furthermore, although other methods, such as ptychography, offer the capabilities of extending beyond these zone‐plate limits, a trade‐off must be balanced between spatial resolution, acquisition time, sample volume, data size, and cost.^[^ ^11^, ^12^ ^]^ Appreciably this is a nontrivial task; consequently, high‐throughput, lab‐based imaging is rarely reported, i.e., the ability to quickly scan a large volume, while retaining sufficient resolution to visualize features of interest. Synchrotron sources produce X‐ray beams of significantly higher flux and brilliance, permitting lower exposure times, and thus faster imaging, however, access is severely limited.^[^ ^13^ ^]^ It is therefore conceivable that a lab‐based system may be positioned in the direct vicinity of a production line, and, provided that the acquisition times can remain in‐line with the production rate, the ability to periodically scan samples may provide an adequate representation of any fluctuations in manufacturing quality. This would be highly beneficial.

Early commercial LIB cathode electrodes were composed largely of cobalt, such as LiCoO_2_ (LCO); however, due to issues such as toxicity and cost, alternatives, such as LiNi_x_Mn_y_Co_z_O_2_ (NMC), are now being explored.^[^ ^14^ ^]^ Currently, Ni‐rich NMC chemistries, such as LiNi_0.8_Mn_0.1_Co_0.1_O_2_ (NMC811) and LiNi_0.6_Mn_0.2_Co_0.2_O_2_ (NMC622) are of particular interest, specifically due to their favorable performance properties for automotive applications. LIB materials present interesting powders for this case study as their manufacture varies considerably, even within the same or very similar chemistries, and can often possess features such as cracks and internal voids beneath the particle surface that 3D X‐ray imaging is ideally placed to assess. Therefore, NMC811 and NMC622 powders were used in this work as exemplar case studies. We report how to prepare powders for microstructural assessment using high‐throughput nano‐CT, providing a platform for 3D QA that outperforms 2D imaging due to the ability to resolve subsurface features; superior statistics due to increased material throughput and access to additional spatial dimensions; and, improved accuracy due to a reduction in perspective/parallax errors.

These techniques have great potential as QA screening procedures, whereby samples can be periodically assessed with minimal acquisition times and computational requirements. Moreover this technique is not limited to battery technologies but may be applied to the broader field of powder metallurgy.

## 2 Results

### 2.1 A Rapid 3D Quality Assurance Method

The two NMC samples were assessed using the optimized preparation and data acquisition methods for maximum image quality, with minimal acquisition time. This preparation involves the production of an approximately monolayer dispersion of particles onto an X‐ray transparent mechanical support. To achieve this, powder is dispersed over an adhesive‐covered surface of Kapton tape and the excess powder is removed by tapping the tape perpendicular to a surface to disperse and settle the powder layer. This is described further within the Experimental Section, including a visual aid of the acquisition procedure.

For each sample, an X‐ray radiograph mosaic was collected without angular rotation, then regions of interest (ROIs) were chosen for 3D analysis. Five volumes were chosen in total, four from the NMC811 and one from the NMC622. The NMC811 ROIs were chosen because they appeared to contain particles of various sizes, i.e., would provide sufficient tests for the versatility of the technique. Whereas the NMC622 ROI was chosen because it contained a large number of particles, therefore would produce the best materials statistics. By employing the novel mounting technique, the average transmission was found to be 8.5% (Figures S1, S2, S3, Supporting Information ). For an exposure time of 1 s and scan duration of approximately 5 min (Figures S4, S5, Supporting Information), the average signal to noise (SNR) for the particles resolved within these tomograms was found to be 10.4, sufficient for the analysis (Figure S6, Supporting Information).

### 2.2 Rapid 3D Quality Assurance of NMC811

**Figure 1:**
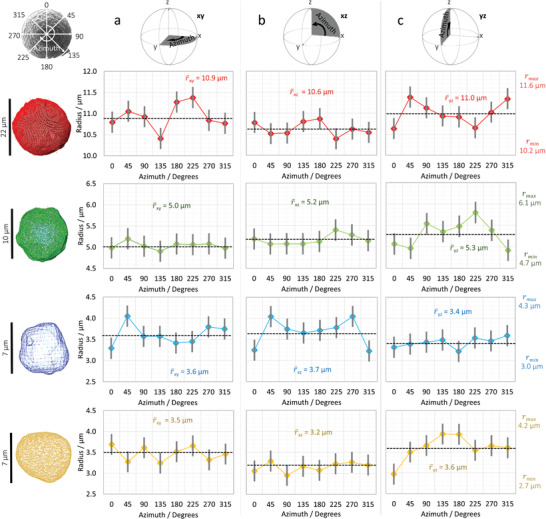
Analyzing single NMC811 particles characterized using 5‐min X‐ray computed tomography (CT). Radii measurements including error bars for the four particles (red, green, blue, and yellow) at eight azimuthal positions for a) the *x*−*y*, b) the *x*−*z*, and c) the *y*−*z* orthogonal planes. All quantifications reported are from the 5‐min X‐ray CT data. Raw values can be found in Table S1 and S2, Supporting Information.

Since the sample was single‐layer, individual particles could easily be extracted for discrete investigation, therefore, four particles (from approx. 160) were chosen for an in‐depth analysis (**Figure** **1**). To improve the statistics and accuracy of a particular quantification, such as the particle radius, values can be quantified from several orientations, reducing the parallax error. For instance, X‐ray CT permits radial measurements to be conducted for various azimuthal angular positions, for several orthogonal planes (i.e., *xy*, *xz*, *yz*), effectively in any orientation. This is not possible with techniques such as scanning electron microscope (SEM) imaging and, although tilting the sample can improve perspective errors, 3D imaging remains superior as 2D imaging cannot reveal the underside of the sample.

Figure 1 presents four NMC811 particles characterized with the high‐throughput X‐ray CT method. The surface meshes for each particle are displayed with accompanying plots of the radii measurements taken at eight azimuthal positions (through 360° at 45° angular increments) displayed for each of the three orthogonal planes: *xy*, *xz*, and *yz* (Figure 1a–c).

**Figure 2:**
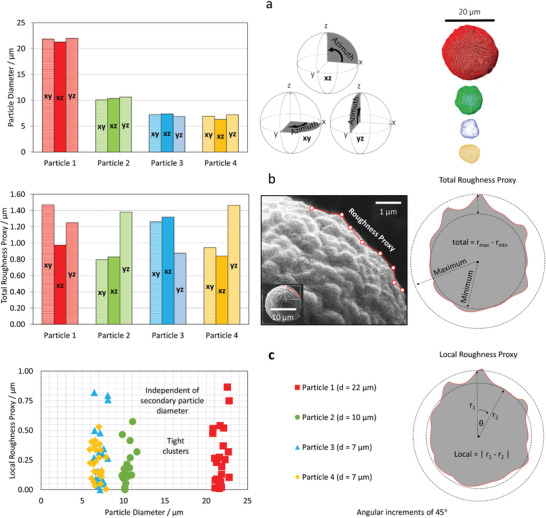
Assessing NMC811 particle diameters and a proxy for surface roughness. a) The particle diameter calculated for each plane (*xy*, *xz*, and *yz*) from an average of the azimuthal radii (i.e., 8 values at 45° increments through 360°). b) The total roughness proxy for each particle with a scanning elecron microscope (SEM) image and illustration for visual aids for real versus proxy surface roughness. c) Local surface roughness proxy, calculated for each particle. All quantifications reported are from the 5‐min X‐ray computed tomography (CT) data. Raw values can be found in Table S1 and S2, Supporting Information.

The radii were averaged through the eight azimuthal positions to produce an average diameter value for each plane (**Figure** **2**a). The SEM imaging (Figure 2b) suggested that the particles are quite spherical in shape, with relatively smooth surfaces, and the X‐ray data generally corroborates this (additional SEM images can be found in the Experimental Section). For each particle, the radius measurements are relatively consistent, regardless of orientation. The average radii values display minimal deviation, i.e., the average values for each orthogonal plane are similar, e.g. for the first particle (red) ${\bar{r}}_{xy}$ = 10.9 µm, ${\bar{r}}_{xz}$ = 10.6 µm, and ${\bar{r}}_{yz}$ = 11.0 µm, averaging 10.8 µm.

These particles are “secondary agglomerates” in that they are composed of many smaller “primary” particles that are on the order of a micron or several hundred nanometers in diameter. This gives rise to surface roughness, as seen in the SEM imaging (Figure 2b). Therefore the variation in radii measurements (i.e., maximum−minimum recorded azimuthal value) was calculated for each plane, and for each particle, as a high‐speed proxy indication of surface roughness (a “roughness proxy”). Secondary particles are assembled from the same primary particles irrespective of size and therefore the surface can be expected to be comparable among all secondary particles. Accordingly this is what is observed in Figure 2b; all particles display a roughness proxy of ≈1 µm, i.e., there is a 1 µm variation between the largest and smallest value of radius obtained. This can also be calculated locally by taking the difference of two azimuthal positions separated by a certain angular increment, in this case 45° (Figure 2c). This further emphasizes the independence between secondary particle size and the degree of surface roughness, as seen through the tightly clustered plots within Figure 2c that extend from minimal variation (i.e., approaching 0 µm) to ≈1 µm, regardless of the particle diameter.

It should, however, be noted that this is only a proxy for roughness and that metrics such as this can possess fractal properties, and are therefore dependent upon the characterization resolution both in terms of the voxel or spatial resolution and the angular increment.^[^ ^15^, ^16^ ^]^ Nonetheless, this is one method that may be applied in order to reduce the necessity for longer duration imaging and intensive computations.

The four particles chosen vary considerably in size; the smallest being ≈7 µm in diameter, and the largest ≈22 µm. This is a demonstration of the versatility of this technique; structures of various shape and size can be successfully characterized in minutes with nanometer scale voxels. Moreover, large volumes of powder can be assessed quickly, allowing the detection of abnormalities. Further statistical benefits of such rapid screening will be considered in the subsequent analysis of the NMC622 powder.

### 2.3 Rapid 3D Quality Assurance of NMC622

**Figure 3:**
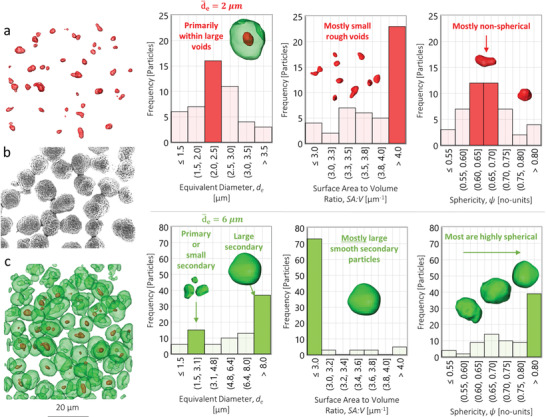
Analyzing single NMC622 particles characterized using 5‐min X‐ray computed tomography (CT). a) internal void space (red) with accompanying structural analysis, b) scanning elecron microscope (SEM) image of the same powder but different region of interest (ROI), for comparison, and c) external particle (green) with accompanying structural analysis.

To demonstrate that the rapid QA method is applicable to other materials and microstructures, we looked at a second type of powder with a different characteristic morphology: NMC622. When inspecting the NMC622 powder using the X‐ray‐CT method (**Figure S7,** Supporting Information), internal voids were observed (**Figure** **3**a) that were not visible under the SEM (Figure 3b). Such morphological features have previously been reported within NMC, however they do not appear within all of the particles; approximately 55% of the particles displayed the internal voids.^[^ ^17^ ^]^

Generally, the internal voids (Figure 3a) do not possess a well‐defined structure: they are small, 2 µm in diameter on average; display a high surface‐area‐to‐volume ratio (SA:V), mostly >4.0 µm^−1^; and are nonspherical, averaging a sphericity of ≈0.65. Whereas the NMC622 particles (Figure 3c) that surround the voids are predominantly large, 8.0 µm in diameter; exhibit a lower SA:V, mostly <3.0 µm^−1^; and are generally highly spherical, mostly sphericity values are >0.8.

The NMC particle and void structures are compared quantitatively in **Table** **1**. Approximately 55% of the NMC622 particles displayed internal voids with ≈4% of the particle volume consumed by this dead space. This is highly important for applications where maximizing volumetric energy density is crucial, e.g. EV range. Assuming that loss of active material equates to loss of drivable range, a net ≈2% dead‐volume (i.e., 4% dead‐volume occurring in 55% of particles) could reduce a 300 mile EV range by 6 miles. Although the reality is more complex, the impact may still be substantial.

**Table 1:** The quantitative information obtained from the NMC622 powder with reference to the two phases of interest: the internal void space and the active NMC material

	Particle	Void
Volume, *V* [µm^3^]	238	9
Surface area, SA [µm^2^]	234	30
Sphericity, *ψ* [no‐units]	0.8	0.7
Surface area to volume ratio, SA:*V* [µm^−1^]	1.7	4.1
Equivalent diameter, *d*_e_ [µm]	6.4	2.4
Percentage of dead volume, *V*_D_ [%]	3.8	
Frequency of dead volumes, *x* in 100 [particles]	55	

Perhaps even more problematic than volumetric density, may be the increased potential for degradation that such internal voids may create. For instance, from an electrochemical perspective, the additional surface area may provide further nucleation sites for various parasitic degradation reactions and structural reordering. Moreover, the mechanical implications may also be multifaceted; voids may act as low‐stress space into which particles may expand during cycling, however may also produce nonuniform stress distribution and microcracking.^[^ ^18^, ^19^ ^]^

## 3 Conclusion

The QA procedures outlined here provide a method for unprecedented material throughput, capable of screening powders in 3D. To the authors’ knowledge, this demonstration on LIB electrode particles presents an order of magnitude time improvement upon the current literature with regards to state of the art 3D imaging of particle microstructures. Consequently, this method is able to produce exceptional material statistics while exposing subsurface detail, with improved accuracy through reduced parallax error.

By applying this method, four volumes of NMC811 powder were analyzed, where each volume was collected in under 5 min. From these volumes, four individual particles of various sizes were chosen for in‐depth analysis. This study provided a fundamental example of one of the benefits of 3D imaging: increased accuracy through reduced parallax error. By analyzing each particle's radius, in the three orthogonal planes, at various azimuthal positions, an indication of the sphericity and surface roughness could be obtained with minimal parallax influence. It was found that, relative to their size, the particles’ radii did not deviate significantly from the average, indicating a relatively high degree of sphericity; however, the undulations in the radii values, which can be used as a proxy for surface roughness, were found to be independent of the particle size, a likely consequence of the particle fabrication, i.e., each of these are secondary particles (on the order of tens of microns), that are composed of many primary particles (on the order of hundreds of nanometers).

To demonstrate sample versatility, a second chemistry was studied: NMC622. Within this batch, a subsurface structural defect was found in the form of internal voids. Approximately 55% of the particles contained these voids which, if electrolyte cannot access, is likely wasted or “dead” volume. Alternatively, these voids may provide additional surface area upon which nucleation points for degradation mechanisms may occur.

Findings such as this may prove hugely valuable for applications within the automotive sector, where optimization in particle morphology, may result in a considerable addition to the drivable range. Future work may look to investigate the manufacturing of coated NMC811 or solid‐state electrolytes whereby, powder analysis with high statistical confidence, may also provide useful input for industrial processes.^[^ ^20^, ^21^ ^]^ Ultimately the methods explored here are applicable to many materials and technological fields that are not limited to Li‐ion, and may be applied to the wider field of powder metallurgy.

## 4 Experimental Section

##### Materials

Two nickel manganese cobalt oxide (NMC) cathode materials were inspected within this work: NMC811 and NMC622, LiNi_0.8_Mn_0.1_Co_0.1_O_2_ and LiNi_0.6_Mn_0.2_Co_0.2_O_2_ respectively, purchased from a commercial supplier (Targray Inc., Kirkland, Canada).

##### Preparation

**Figure 4:**
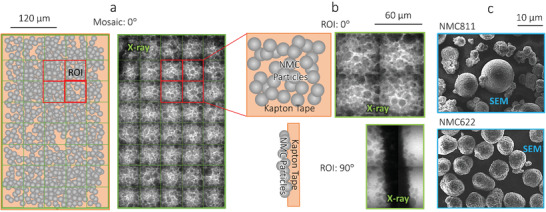
Single‐layer powder mounting for rapid 2D and 3D quality assurance of powders. a) A front‐on illustration and X‐ray radiograph at 0° and 90° (w.r.t. the X‐ray beam path) for b) an region of interest (ROI) visualized within a larger 40‐image mosaic, with c) accompanying scanning elecron microscope (SEM) image of the NMC811 and NMC622 powders.

In an effort to optimize the aforementioned trade‐off between resolution, acquisition time, sample volume, data size, and cost, a novel mounting method was proposed. For the best image quality, image contrast should be maximized by optimizing the X‐ray transmission.^[^ ^22^ ^]^ A 5.4 keV incident X‐ray beam would result in optimum X‐ray transmission (≈14%) when passing through approximately 42 µm of NMC811 (Figure S3, Supporting Information). Moreover, observations via SEM imaging suggested that the particles within the powder vary between 5 and 30 µm in diameter (**Figure** **4**), therefore it was concluded that a single‐layer of powder, mounted onto of a highly x‐ray transparent mechanical support would produce the optimal imaging conditions. Optimal acquisition time was dependent largely on the exposure time and number of projections; by applying a high pixel binning, a large sample volume could be analyzed while significantly reducing the number of projections required, but at the expense of spatial resolution. This work used a square 1024 × 1024 pixel detector with a 64 × 64 µm field of view (FOV). By employing a binning of 8 the optimum number of projections could be reduced an order of magnitude, to 196 (from 1571 for bin = 1). In practice, due to image processing and the lack of useful material at the outermost voxels of a tomogram, significantly fewer projections were often used, consequently 141 projections per tomogram were employed. As mentioned, increasing the binning comes at a loss of the spatial resolution, due to a larger pixel size, here 512 nm; however, for high‐speed analysis and features on the order of tens of microns, this remains sufficient. It should however be noted that the optimum conditions, thus sample thickness, were dependent upon the sample chemistry and incident beam energy.^[^ ^23^ ^]^ With the sample preparation and data acquisition optimized for maximum image quality, with minimal acquisition time, a 40‐image 2D radiograph mosaic was collected, from which four ROIs were chosen (Figure 4a) and a tomography scan was conducted (Figure 4b). These regions were chosen because they appeared to contain particles of various sizes, i.e., would provide sufficient tests for the versatility of the technique.

##### X‐Ray Imaging

**Figure 5:**
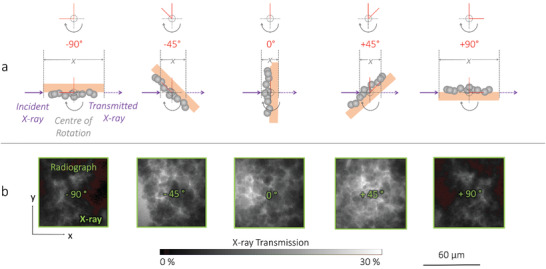
A method for 5‐min X‐ray computed tomography (CT) of powders. a) A top‐down illustration of the sample rotation during tomography acquisition of the region of interest (ROI) from ‐90° to +90°; and, b) X‐ray radiographs of the ROI from −90 ° to +90 °, with accompanying transmission scale.

All imaging was conducted using a lab‐based Zeiss Xradia 810 Ultra X‐ray instrument (Carl Zeiss Inc., CA, USA) employing a quasi‐monochromatic 5.4 keV, parallel‐beam geometry coupled to a Fresnel zone plate focusing architecture. Radiography was conducted using a pixel binning of 8, producing an effective pixel size of 512 nm. Each radiograph image was collected using an exposure time of 1 s and represented an area with a FOV of 64 × 64 µm. The FOV can be repeated in a mosaic pattern to increase the imaged area; for this work 40 images were collected in a 5 × 8 grid and stitched into a large map, ≈160 000 µm^2^ in total. This grid took under 1 min to produce and capture approximately 30 particles per image, over a thousand in total. Each image contained information in a grayscale format, and was ≈30 KB in size. By applying a binning of 8, sufficient SNR could be obtained using an exposure time of 1 s and 141 projections, i.e., collecting enough radiograph projections for a total acquisition time of under 5 min (including reference images and motor movements) (**Figure** **5**). The radiographs could then be reconstructed to produce a 64 × 64 × 64 µm, ≈200000 µm^3^, 3D grayscale volume, ≈4 MB in size and with an isotropic 512 nm voxel size. All reconstructions were achieved using commercial software (“Reconstructor Scout‐and‐Scan”, Carl Zeiss Inc., CA, USA).

##### SEM Imaging

For comparison and corroboration, SEM imaging was conducted for each of the cathode powders. To do this, a small amount of powder was sparsely deposited onto carbon tape that was adhered to small stubs, ≈1 cm in diameter. Imaging was then conducted using an EVO MA 10 SEM (Carl Zeiss, USA) with an accelerating voltage of 15 kV and working distance of 8.0 mm.

##### Data Analysis

All data processing (post‐reconstruction) was conducted using Avizo Fire software (Avizo, Thermo Fisher Scientific, Waltham, MA, USA). Volume renderings of the raw grayscale data were carried out without segmentation but with a threshold applied so that all grayscale values above a certain value appear transparent when viewed in 3D. For true segmentation of the raw grayscale data into binary matrices, i.e., into solid and pore phases, grayscale thresholds were applied. Tertiary segmentation was also required for some datasets that included internal (as well as external) voids, requiring a flooding segmentation algorithm. The flooding algorithm was as follows: all voxels containing empty space were allocated one value and all voxels containing NMC material were allocated a second value, then a space external to the particles was selected and all voxels that were in direct contact with that space were allocated a third value, resulting in a tertiary dataset of particles, internal, and external voids. For all single particle segmentations, a spherical region was defined in order to completely encompass the particle, then all data external to this sphere were disregarded for forthcoming computations. For the binary and tertiary data, surface generations provide a better visual representation of the particles and voids, therefore, where stated, surfaces were generated for visual representation; however, all quantitative analysis was conducted using the cubic data, i.e., did not include the data smoothing inherent in the surface algorithms. Compositional analysis was completed by the summation of all segmented voxels within the volume of interest. For the quantification of the NMC811 particle radii, eight 2D measurements were made using Avizo for each of the three orthogonal planes: *xy*, *xz*, and *yz*; all raw data can be found in the Supporting Information. To assess the sphericity of particles, equivalent volumes and surface areas were calculated from the averaged measured values for the radius for each particle. These were then compared to the measured volume and surface area of each particle. Where error bars are presented, the error was calculated from the imaging resolution. An example calculation for a random shape is described visually in Figure S8, Supporting Information.

##### Trade‐Offs

This work optimized the trade‐off between memory size, resolution, acquisition time and operational cost while maintaining the same volume analyzed. In short, approximately a two order of magnitude memory size and cost reduction might be achieved through a spatial resolution sacrifice of 150 to 512 nm. More information can be found within Tables S3, S4, and S5, and Figures S9 and S10, Supporting Information.

## Conflict of Interest

The authors declare no conflict of interest.

## Author Contributions

T.M.M.H. and P.R.S. conceived the mounting and imaging method. T.M.M.H. conducted all X‐ray imaging. A.V.L. conducted all SEM imaging. A.S.L. and C.T. aided T.M.M.H. in the construction of the article. M.D.R.K. aided in the data extraction and transmission calculations. R.J. provided the powder. R.J., D.J.L.B. and P.R.S. directed the project and provided guidance throughout, including all sourcing of equipment and financial support. All authors oversaw and reviewed the writing of the manuscript.

## Supporting information

Supporting Information
