# Supplementary material for: Resolving Li‐Ion Battery Electrode Particles Using Rapid Lab‐Based X‐Ray Nano‐Computed Tomography for High‐Throughput Quantification
Source: Adv Sci (Weinh). 2020 Apr 30;7(12):2000362. doi: 10.1002/advs.202000362 (PMC7312274; doi:10.1002/advs.202000362)
Supplement: Supplementary file 1 — Supporting Information [file ADVS-7-2000362-s001.pdf]

Copyright WILEY-VCH Verlag GmbH & Co. KGaA, 69469 Weinheim, Germany, 2018.

Supporting Information

Resolving Li-ion Battery Electrode Particles Using Rapid Lab-based X-ray Nano-Computed Tomography for High Throughput Quantification: Supporting Information

*Thomas M. M. Heenan, Alice V. Llewellyn, Andrew S. Leach, Matthew D. R. Kok, Chun Tan, Rhodri Jervis, Dan J.L. Brett and Paul R. Shearing**

Electrochemical Innovation Lab, Department of Chemical Engineering, UCL, London WC1E 7JE, U.K.

The Faraday Institution, Quad One, Harwell Science and Innovation Campus, Didcot, OX11 0RA, U.K.

E-mail: P.Shearing@ucl.ac.uk

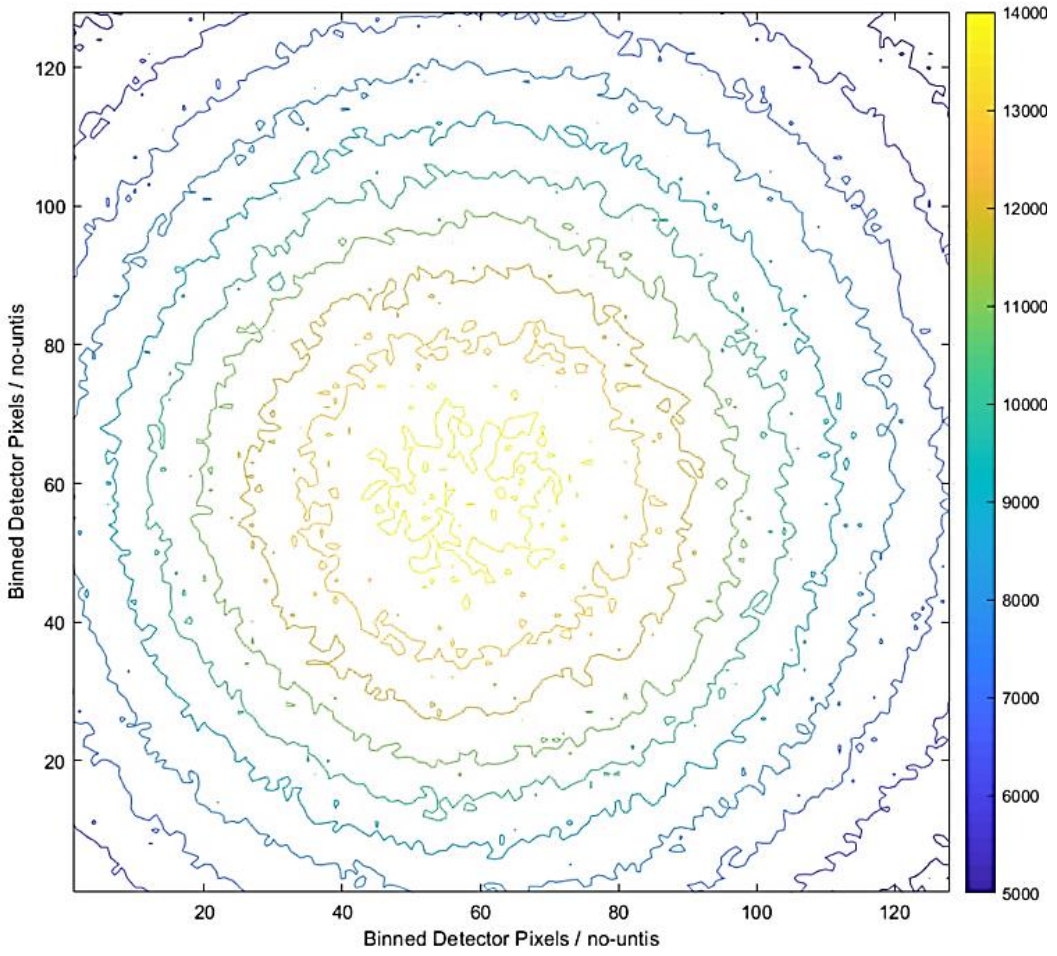

Figure S1. Example 'bright' X-ray radiograph for transmission calculations. Color bar units in detector counts. This image was acquired using a binning of 8 and an exposure time of 1 second onto a 1024 x 1024 pixel detector.

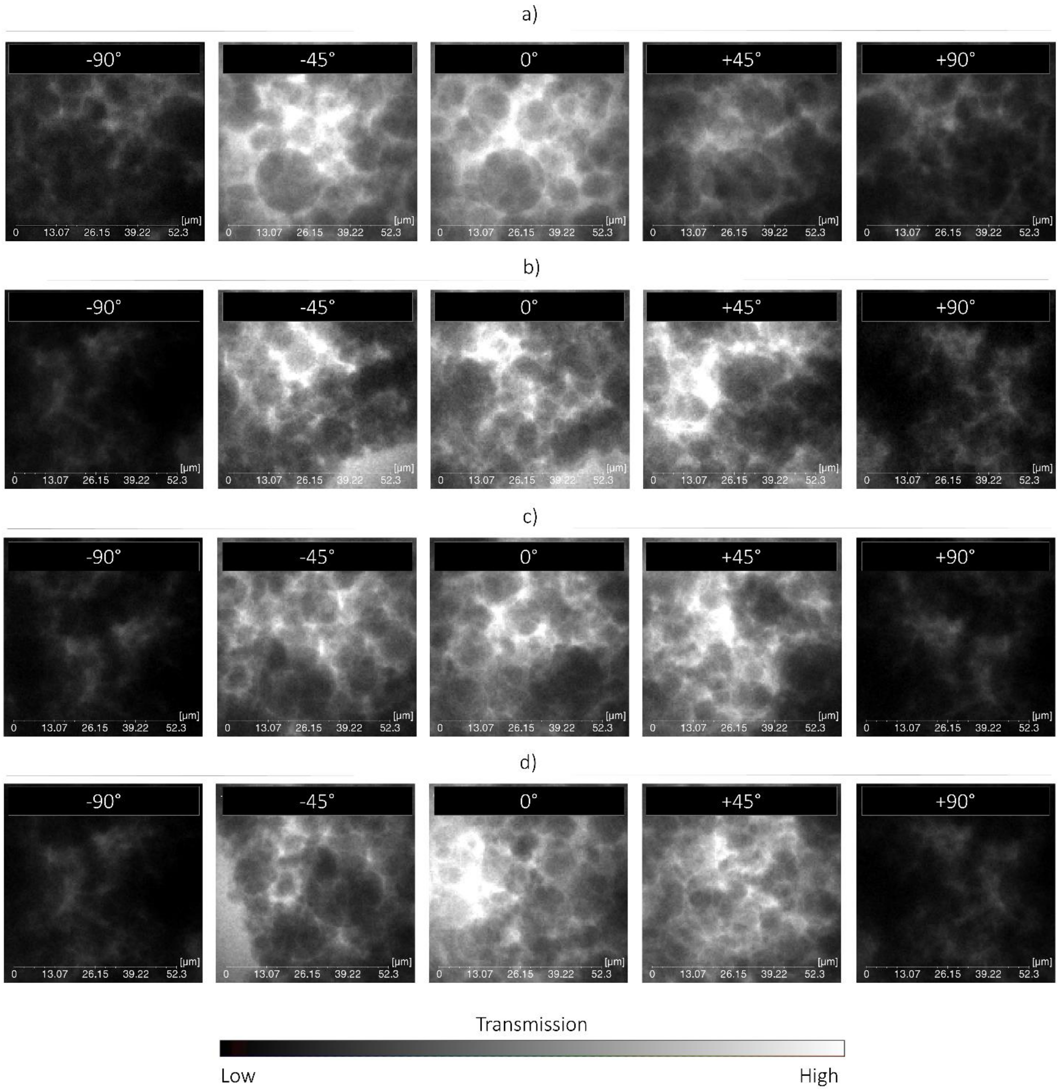

Figure S2. Reference-corrected 2D radiograph images of the four regions within the NMC811. A, b, c and d, taken at 90°, -45°, -0°, +45° and +90, on the lab-based Xradia 810 ULTRA X-ray instrument, with an exposure time of 1 s per radiograph and 141 projections over the 180° range. Color bar represents X-ray transmission whereby low = 0 % and high = 30 %.

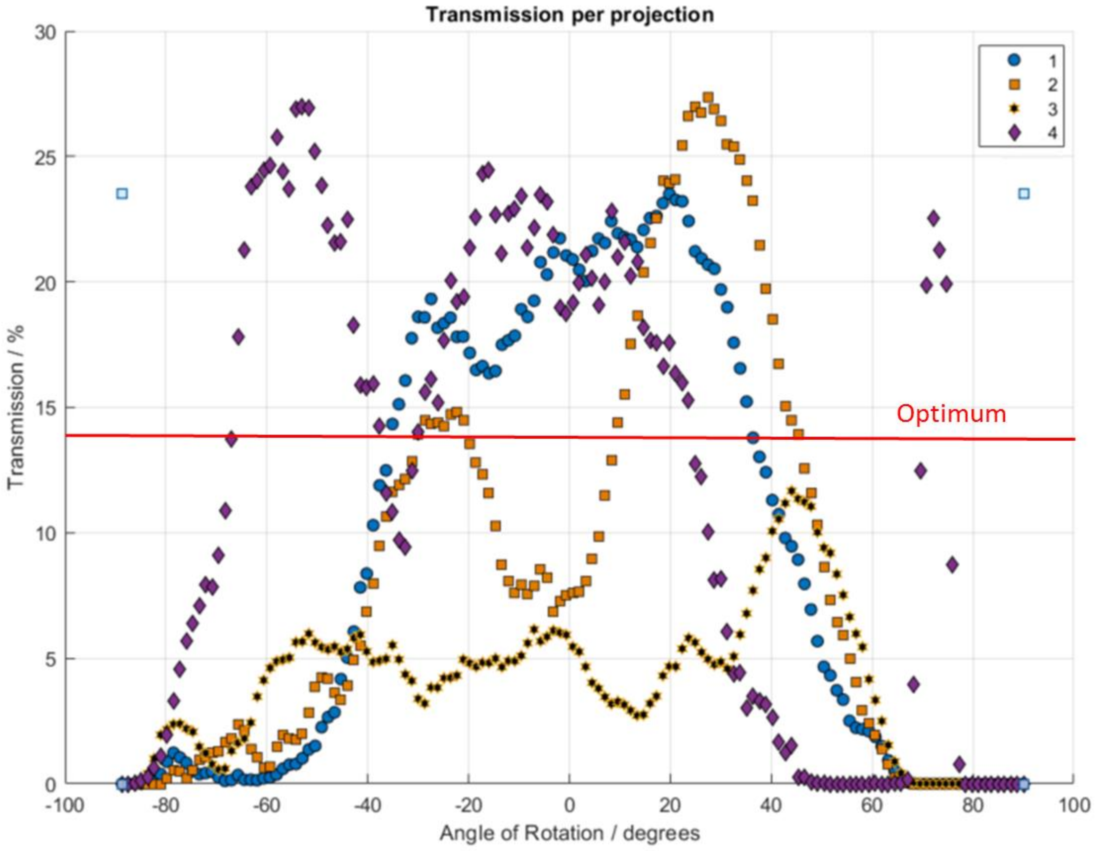

Figure S3. Transmission for the NMC811 X-ray radiogrpah sets. The mean transmission per projection for the four NMC811 tomography scans through the 141 angular projections from – 90° to 90°.

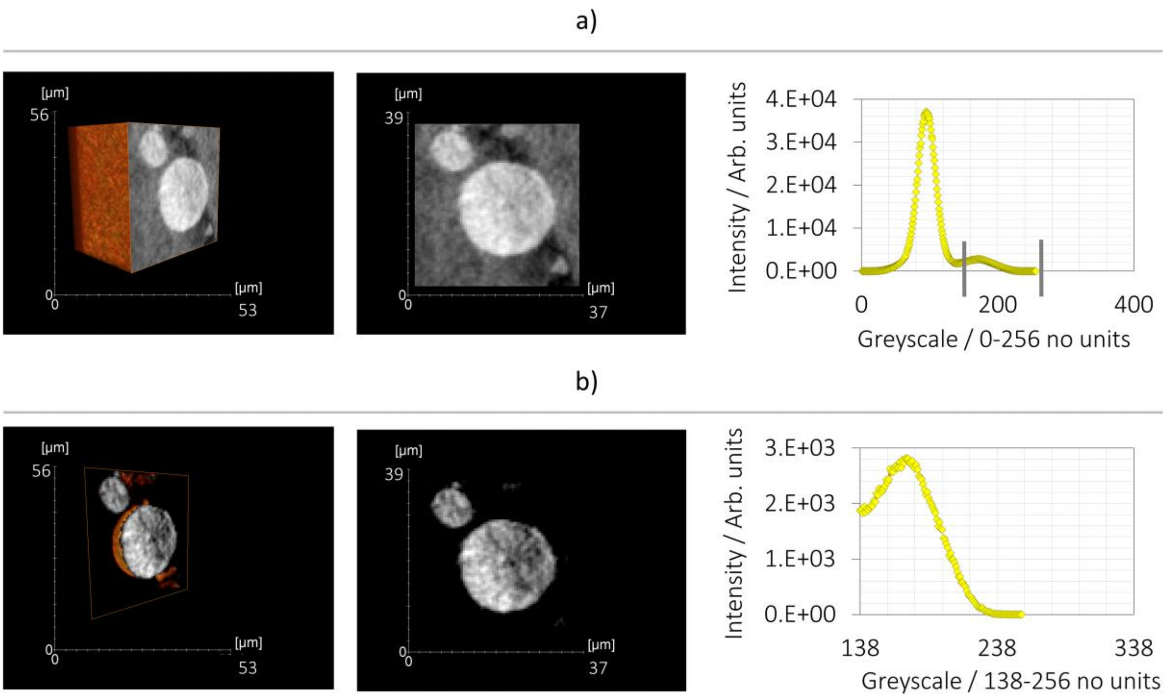

Figure S4 An example of 3D greyscale segmentation for NMC811. An example of the 3D segmentation procedure: a) a full greyscale range and b) a cropped greyscale range.

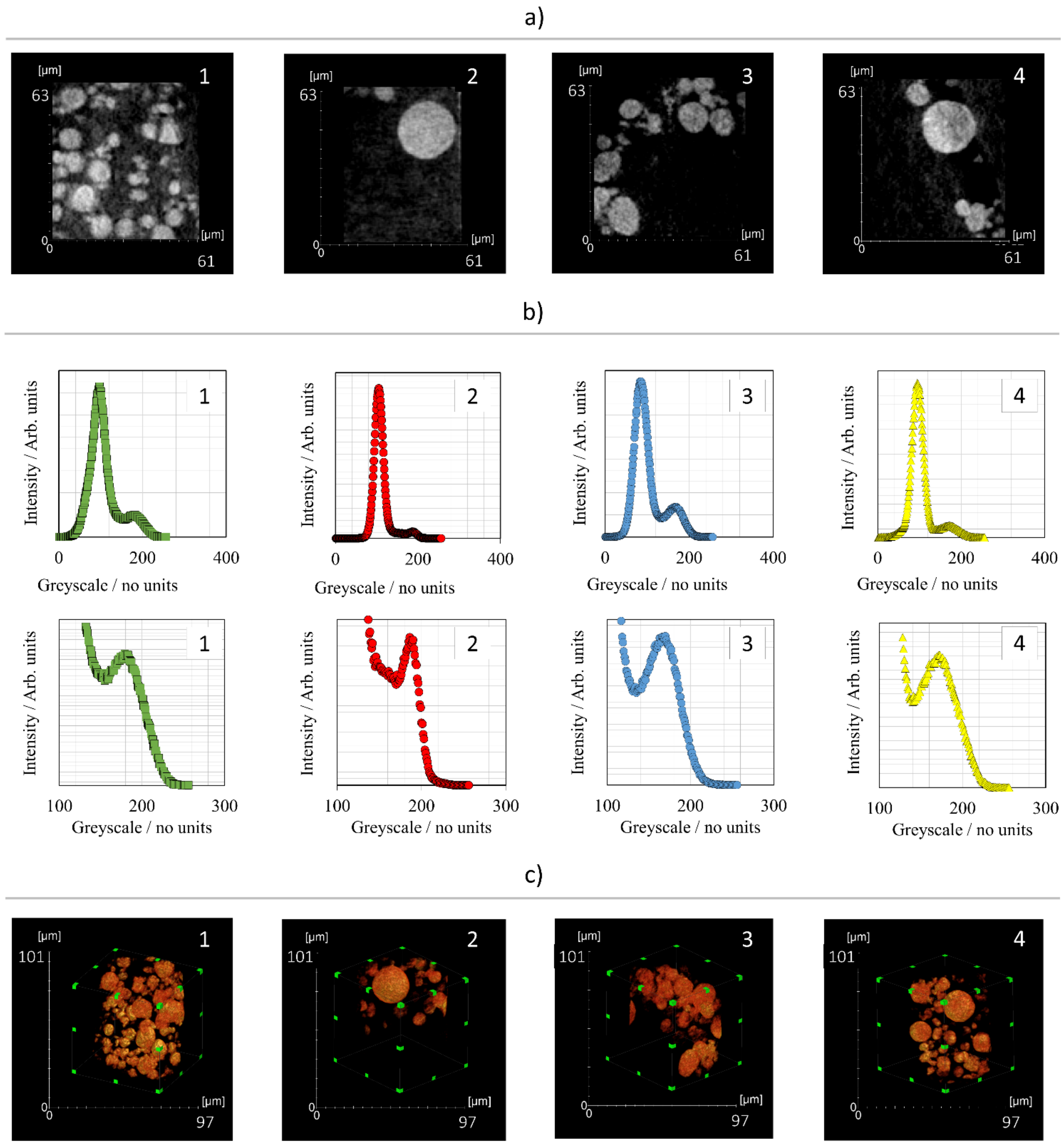

Figure S5. Four rapid-CT scans of the NMC811 particles. a) raw unfiltered ortho-slices taken from each of the tomograms, b) accompanying greyscale histograms with (top row) full and (bottom row) cropped greyscale range, and c) 3D volume renders based upon the greyscale crop above them.

Equation S1 was used to define the SNR per reconstructed tomogram slice. Whereby the signal, $\bar{I}$, was defined as the greyscale values for the particles. This was done so using a mask to replace all pore voxels with non-number values.

$$SNR = \frac{\bar{I}}{\sigma_g} \quad \text{(S1)}$$

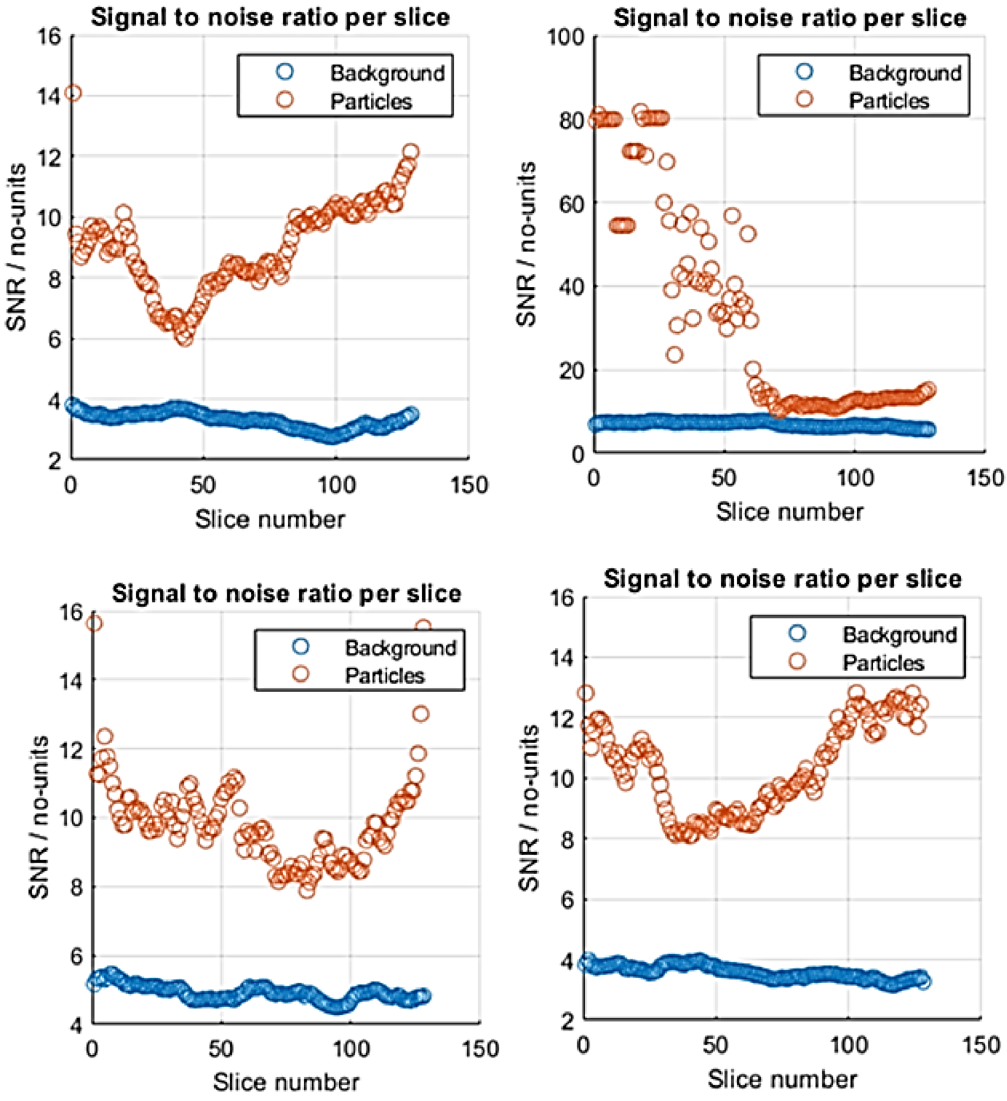

Figure S6. Signal to noise calculations for four NMC811 data sets displayed with resepct to slice number within the tomogram.

Table S1. Local quantifications from the NMC811 powder. Measurements of the particle radii taken at the eight locations for each of the three orthogonal axis: xy, xz and yz, 24 total measurements, for each of the four particles, obtained using the RAPID-CT method.

Angle / Degrees	Particle 1			Particle 2			Particle 3			Particle 4		
	xy	xz	yz	xy	xz	yz	xy	xz	yz	xy	xz	yz
0	10.80	10.78	10.64	4.99	5.19	5.07	3.29	3.25	3.32	3.69	3.06	2.97
45	11.06	10.52	11.39	5.20	5.07	4.97	4.05	4.04	3.39	3.28	3.29	3.50
90	10.93	10.53	11.13	5.03	5.08	5.54	3.57	3.75	3.44	3.61	2.95	3.66
135	10.41	10.81	10.94	4.91	5.08	5.36	3.57	3.66	3.49	3.25	3.17	3.94
180	11.28	10.87	10.92	5.08	5.13	5.48	3.42	3.72	3.22	3.52	3.07	3.93
225	11.38	10.40	10.66	5.06	5.40	5.80	3.45	3.78	3.54	3.66	3.23	3.55
270	10.84	10.63	11.03	5.08	5.29	5.39	3.80	4.05	3.47	3.32	3.26	3.66
315	10.77	10.55	11.35	4.98	5.15	4.92	3.75	3.23	3.59	3.46	3.20	3.61

Table S2. Bulk quantifications from the NMC811 powder. The minimum, average, maximum and range of the radii values measured from the four particles using the RAPID-CT method.

	Particle 1			*Particle 2*			*Particle 3*			*Particle 4*		
	xy	xz	yz	xy	xz	yz	xy	xz	yz	xy	xz	yz
Minimum	10.4	10.4	10.6	4.9	5.1	4.9	3.3	3.2	3.2	3.2	3.0	3.0
Average	10.9	10.6	11.0	5.0	5.2	5.3	3.6	3.7	3.4	3.5	3.2	3.6
Maximum	11.4	10.9	11.4	5.2	5.4	5.8	4.1	4.0	3.6	3.7	3.3	3.9
Range	0.5	0.2	0.4	0.1	0.2	0.4	0.4	0.4	0.2	0.2	0.2	0.5

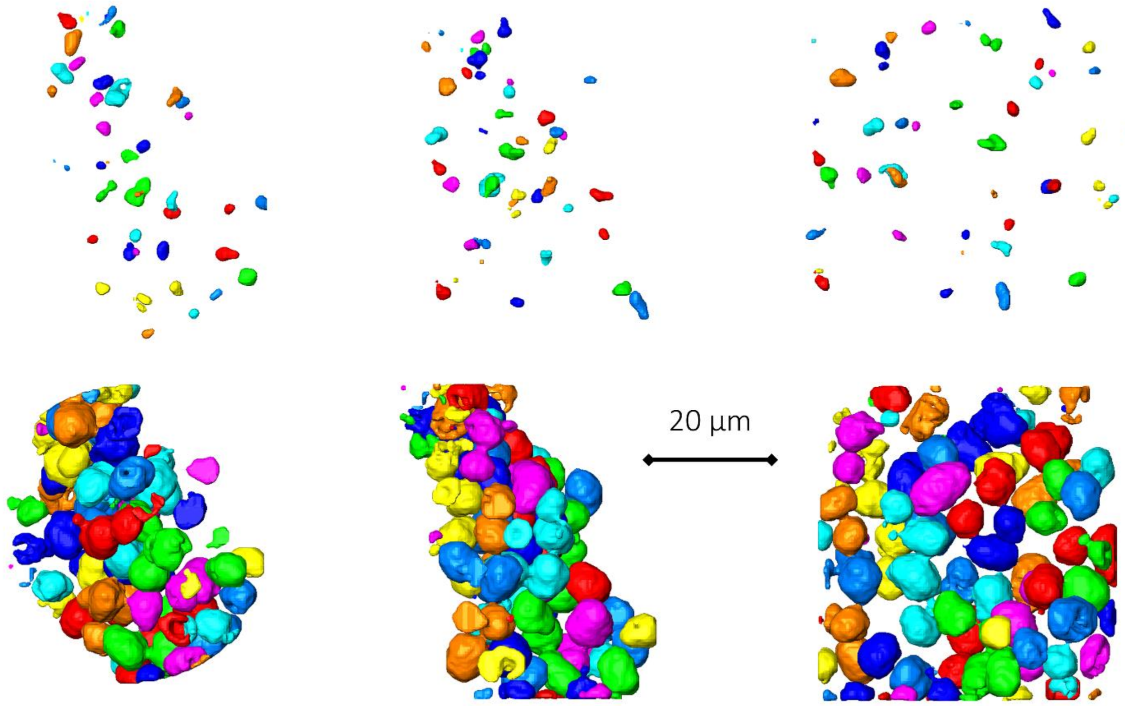

Figure S7. NMC622 segmentation and separation. Segmented NMC622 particles that have been separated into two phase: top row) internal voids, and bottom row) active NMC622 material. Then each particle has been separated for individual analysis and color coordinated.

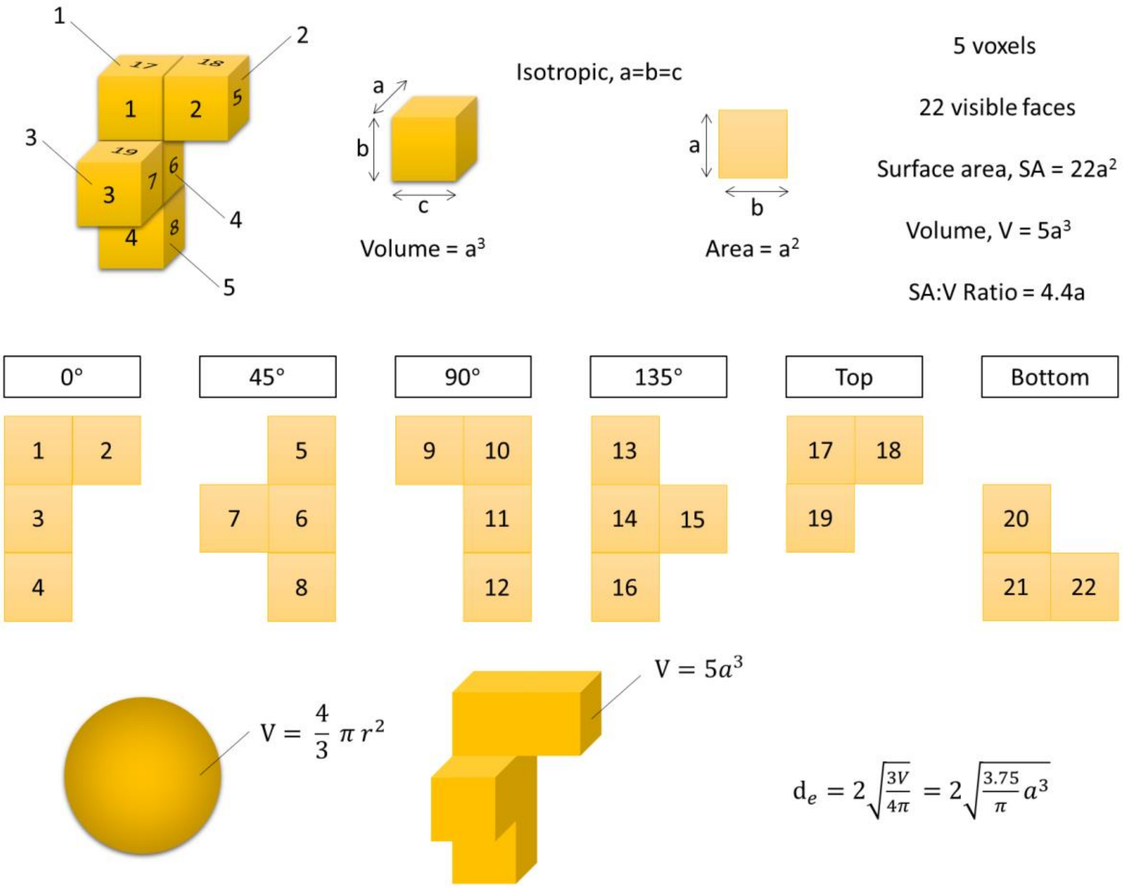

Figure S8 Example 3D quantifications on a random shape whereby five voxels constitute the 3D volume which has 22 visible faces.

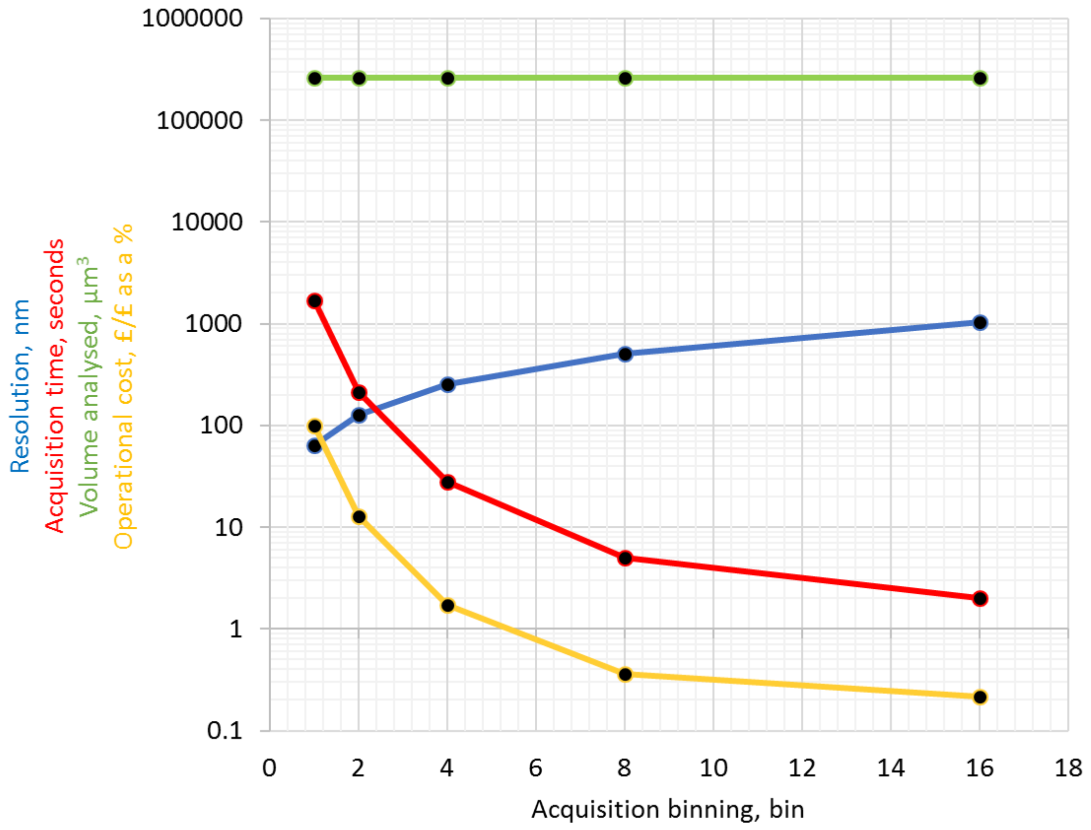

Figure S9 Data acquisition trade-off analysis for: volume analysed, resolution, acquisition time and operational cost, all with respect to binning.

Table S3 Data acquisition trade-off analysis for: volume analysed, resolution, acquisition time and operational cost, all with respect to binning.

**Binning (memory)**	-	1	2 (1000 MB)	4	8 (4 MB)	16
**Spatial resolution**	nm	150	150	256	512	1024
**Voxel size**	nm	64	128	256	512	1024
**Projections**	-	1571	785	393	196	98
**Exposure time**	sec	64	16	4	1	0.3
**Acquisition time**	min	1678	211	28	5	2

**Volume analysed**	μm^3	262144	262144	262144	262144	262144
**Field of view**	µm	64	64	64	64	64
**Detector size**	pixels	1024	1024	1024	1024	1024
**Effective detector size**	pixels	1024	512	256	128	64
**Operational cost**	£/£ as a %	100.00	12.66	1.72	0.36	0.21

The following table can be used as an indication of the approximate time breakdown for this method compared to traditional methods.

Table S4 A comparison of the various time requirements in the analysis of particles.

	**Rapid method**	**Traditional methods**+
Sample preparation	Under 10 minutes	Tens of minutes or hours
Acquisition	Under 5 minutes	Several hours or days
Reconstruction	Under 1 minute	Tens of minutes
Manual segmentation	~20 minutes	~20 minutes*
Analysis	~30 minutes	~30 minutes*
Approx. turnaround time	Hours<br>(Under an hour if automated)	Days<br>(Still days even if automated)

+Traditional methods being laser lathe or focused ion beam lift-out

*Segmentation and analysis will be computationally slower with larger datasets but not vastly.

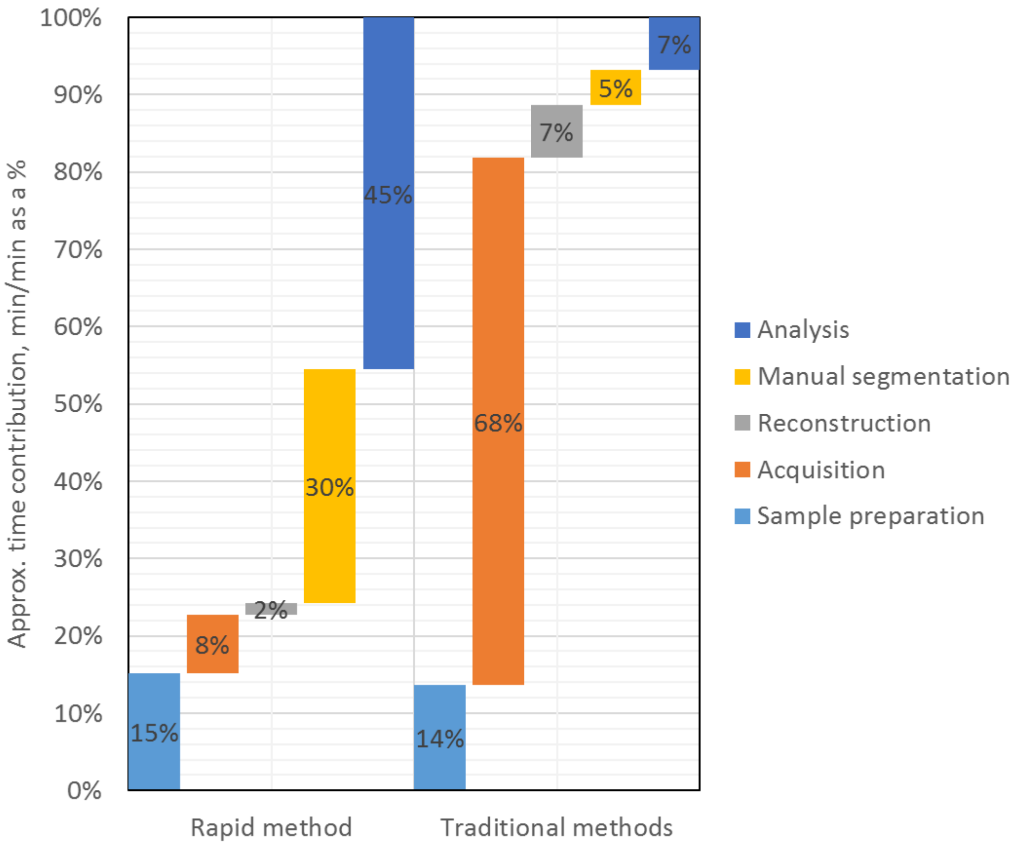

Figure S10 A comparison of the approximate contribution of the sample preparation, acquisition, reconstruction, segmentation and analysis to the total time, compared between this 'rapid' method and traditional methods.

The approximate times used to generate Figure S10 are noted in the following Table S4.

Table S4 Example time requirements in the analysis of particles displayed in minutes.

	**Rapid method**	**Traditional methods**
Sample preparation	10	60
Acquisition	5	300
Reconstruction	1	30
Manual segmentation	20	20
Analysis	30	30
